# Identification and Biological Evaluation of a Novel Small-Molecule Inhibitor of Ricin Toxin

**DOI:** 10.3390/molecules29071435

**Published:** 2024-03-22

**Authors:** Xinran Yang, Aili Wei, Xiyuan Cao, Zicheng Wang, Hongzhi Wan, Bo Wang, Hui Peng

**Affiliations:** 1 State Key Laboratory of Toxicology and Medical Countermeasures, Institute of Pharmacology and Toxicology, Academy of Military Medical Sciences, Academy of Military Sciences, Beijing 100850, China; yxr200621t143@163.com (X.Y.);; 2 Department of Operational Medicine, Institute of Environmental and Operational Medicine, Academy of Military Medical Sciences, Academy of Military Sciences, Tianjin 300050, China

**Keywords:** ricin, small-molecule inhibitor, antidote, molecular docking

## Abstract

The plant-derived toxin ricin is classified as a type 2 ribosome-inactivating protein (RIP) and currently lacks effective clinical antidotes. The toxicity of ricin is mainly due to its ricin toxin A chain (RTA), which has become an important target for drug development. Previous studies have identified two essential binding pockets in the active site of RTA, but most existing inhibitors only target one of these pockets. In this study, we used computer-aided virtual screening to identify a compound called RSMI-29, which potentially interacts with both active pockets of RTA. We found that RSMI-29 can directly bind to RTA and effectively attenuate protein synthesis inhibition and rRNA depurination induced by RTA or ricin, thereby inhibiting their cytotoxic effects on cells in vitro. Moreover, RSMI-29 significantly reduced ricin-mediated damage to the liver, spleen, intestine, and lungs in mice, demonstrating its detoxification effect against ricin in vivo. RSMI-29 also exhibited excellent drug-like properties, featuring a typical structural moiety of known sulfonamides and barbiturates. These findings suggest that RSMI-29 is a novel small-molecule inhibitor that specifically targets ricin toxin A chain, providing a potential therapeutic option for ricin intoxication.

## 1. Introduction

Ricin is derived from the seeds of the castor bean plant and just 1.78 milligrams can be fatal to adults [1]. Due to its accessibility, simple extraction process, and high stability, the toxin has become a worldwide concern regarding potential bioterrorism attacks and political assassinations [2,3]. Furthermore, as a common oilseed crop, castor seeds are widely cultivated around the world, and the ingestion of castor seeds often leads to intoxication incidents [4]. However, clinical treatment for ricin poisoning still mainly focuses on symptom relief [5], and there is an urgent need to develop targeted therapeutics against ricin.

Ricin is classified as a type 2 ribosome-inactivating protein (RIP) and consists of a catalytic subunit (ricin toxin A chain (RTA)) and a lectin subunit (ricin toxin B chain (RTB)) connected by a disulfide bond [6]. Upon internalizing into the eukaryotic cell and reverse-transporting to the endoplasmic reticulum (ER), RTA and RTB dissociate [7]. Subsequently, RTA, with its highly specific *N*-glycosidase activity, targets and removes the second adenine nucleotide of the conserved GAGA tetraloop of 28S eukaryotic ribosomal RNA (rRNA) in the cytoplasm, thereby inhibiting protein synthesis and elongation [8,9]. RTA can irreversibly depurinate and destroy up to 1500 ribosomes per minute, rapidly halting cellular protein synthesis, and ultimately leading to cell death [10].

Compared to neutralizing antibodies that detoxify only before ricin enters cells [11], and antagonist peptides that are easily degraded in the body and not suitable for oral administration [12,13], small-molecule inhibitors have many advantages. They can act both before and after ricin enters the cells and are less susceptible to changes in the pH level inside the body and degradation [13]. Pteroic acid (PTA) is one of the most widely studied ricin inhibitors [14]. However, its poor solubility limits its development and application as a lead compound, and research on PTA derivatives and analogues is ongoing [15,16,17].

The active site of RTA consists of two binding pockets: a primary pocket that is specific to the adenine substrate, and a slightly larger secondary pocket separated by the side chain of tyrosine 80. The existence of this secondary pocket has been confirmed by the X-ray structure of the RTA–compound complex, indicating that compounds that enter this pocket could induce conformational changes in the primary pocket of active site and affect its activity, leading to antagonism [14,18]. Therefore, it may be essential to target both pockets simultaneously to obtain more effective small-molecule RTA inhibitors [19]. In this study, we discovered a novel compound named RSMI-29 through rational virtual screening, which may target both active pockets of RTA. A biological evaluation of RSMI-29 revealed its effectiveness against protein synthesis inhibition, depurination activity, and cytotoxicity induced by PTA/ricin in vitro, as well as its ability to protect mice from ricin attack in vivo.

## 2. Results

### 2.1. Discover Potential RTA Inhibitors by Virtual Screening

**Figure 1:**
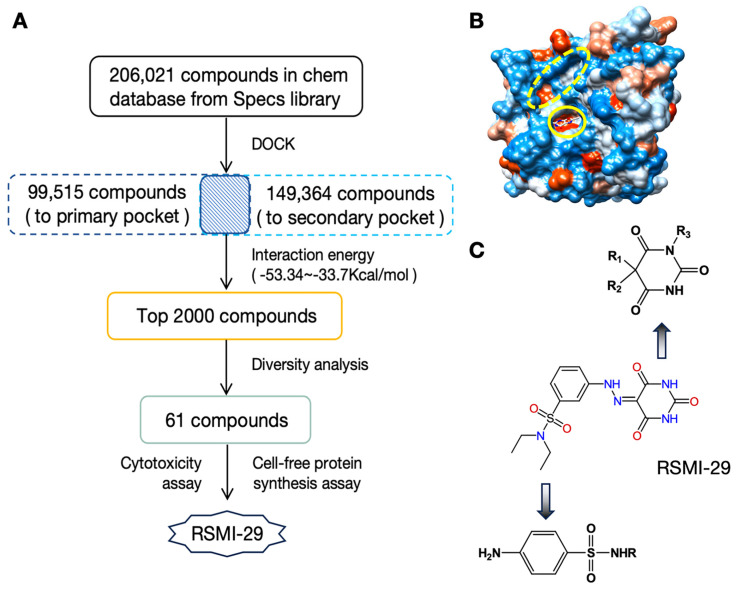
Virtual screening identified candidate RTA inhibitor. (**A**) Virtual screening process of candidate RTA inhibitors. (**B**) Structure diagram of RTA with the primary pocket (solid line) and the secondary pocket (dashed line). (**C**) The chemical structure of compound RSMI-29, *N*,*N*-diethyl-3-(2-(2,4,6-trioxotetrahydropyrimidin-5(2*H*)-ylidenehydraziny)benzenesulfonamide and its drug-like analysis.

As shown in Figure 1A, a comprehensive approach based on virtual screening was utilized to discover novel small-molecule inhibitors of RTA. A database search was carried out on a database containing approximately 206,000 commercially available compounds from specs library (www.specs-group.com accessed on 14 June 2008), which was converted to 3D MOL2 format using in-house procedures [20,21]. The PDB entry 1BR6 [14,22] of RTA in complex with PTA was retrieved from the Protein Data Bank. The primary and secondary pockets of RTA, depicted in Figure 1B, were chosen for docking. Docking results revealed 99,515 molecules binding to the primary pocket of RTA and 149,364 molecules binding to the secondary pocket. The top 2000 compounds with the highest energy scores for both active sites were then selected for further diversity analysis. Sixty-one compounds were tested for biological activity based on the well-known “Rule-of-5” standards and commercial availability. Finally, as shown in Figure 1C, compound No. 29 (named RSMI-29) was identified as the most effective compound in vitro. It is worth noting that RSMI-29 has a typical structural moiety of known sulfonamides and barbiturates, indicating that it has good drug-like properties.

### 2.2. RSMI-29 Inhibits the Cytotoxicity of RTA/Ricin

**Figure 2:**
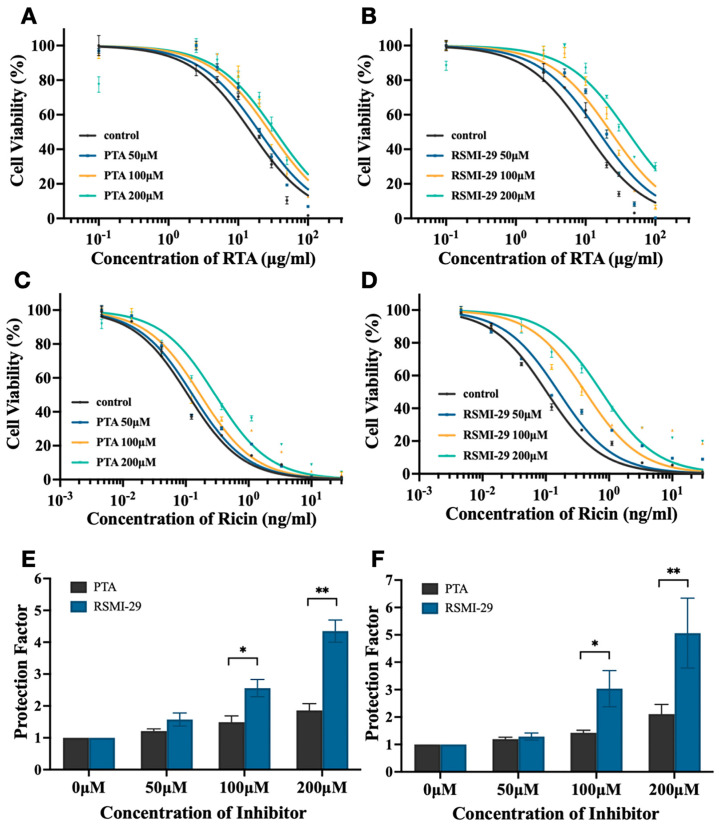
RSMI-29 inhibits cytotoxicity of RTA/ricin. (**A**,**B**) SP2/0 cells were treated with various concentrations of RTA in the absence or presence of different concentrations (50, 100, and 200 μM) of PTA (**A**) or RSMI-29 (**B**) for 24 h followed by CCK8 assay, where 0.1% DMSO was used as negative control. PTA was selected as positive control. The data are representative of three independent experiments. (**C**,**D**) SP2/0 cells were treated with various concentrations of ricin in the absence or presence of different concentrations (50, 100, and 200 μM) of PTA (**C**) or RSMI-29 (**D**) for 24 h, followed by CCK8 assay. The data are representative of three independent experiments. (**E**,**F**) Protection factor of RSMI-29 and PTA in SP2/0 cells treated with RTA (**E**) or ricin (**F**). Protection factor was calculated as EC50_toxin+drug_/EC50_toxin_ (control) ratio. The data shown are mean ± SEM (* *p* < 0.05, ** *p* < 0.01 RSMI-29 group vs. PTA group, n = 3).

The inhibitory effect of RSMI-29 on RTA or ricin-induced cytotoxicity was evaluated by in vitro cellular assays. As shown in Figure 2A,B, the positive controls PTA and RSMI-29 exhibited significant antagonistic effects on cytotoxicity induced by RTA, in a dose-dependent manner. A similar trend was observed in the experiment of ricin (Figure 2C,D). Notably, RSMI-29 showed higher potency against RTA/ricin than PTA, as depicted in Figure 2E,F, where the protection factor of RSMI-29 was over two times higher than that of PTA at the concentration of 200 μM. These results suggest that RSMI-29 effectively attenuates ricin-induced cytotoxicity in a dose-dependent manner and may be superior to the positive control PTA.

### 2.3. RSMI-29 Inhibits the Biological Activity of RTA/Ricin In Vitro

**Figure 3:**
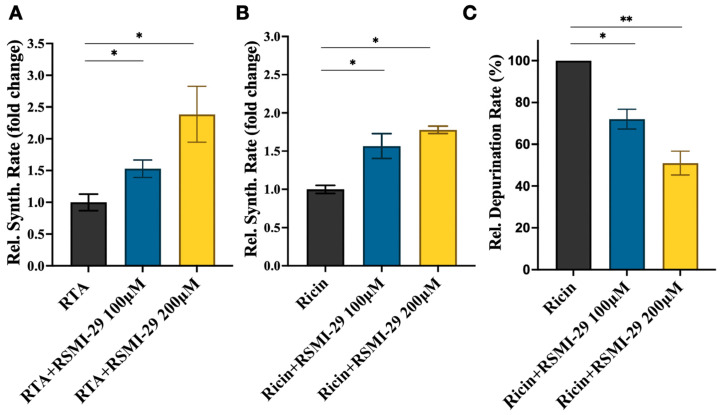
RSMI-29 attenuates RTA/ricin-induced protein synthesis inhibition and rRNA depurination. (**A**,**B**) Rabbit reticulocyte lysate protein synthesis systems containing 20 μg/mL RTA (**A**) or 100 ng/mL ricin (**B**) were treated with solvent DMSO and 100 or 200 μM RSMI-29. After incubation at 30 °C for 90 min, the rate of luciferase protein synthesis was measured. The protein synthesis rate in the RTA/ricin DMSO control group was set as 1, and the relative protein synthesis rate for each treatment group was expressed as mean ± SEM (* *p* < 0.05 vs. RTA/ricin DMSO control group, n = 3). (**C**) Cells were co-incubated with RSMI-29 (100 and 200 μM) and 10 ng/mL ricin for 24 h. RNA was extracted, and the relative level of depurinated rRNA was detected by RT-PCR. The depurination rate of the ricin control group was set as 100%; then, the relative depurination rates for other groups were calculated and presented as mean ± SEM (* *p* < 0.05, ** *p* < 0.01 vs. ricin control group, n = 3).

The rabbit reticulocyte lysate protein synthesis system was employed to investigate the restorative effect of the compound on protein synthesis inhibition induced by RTA/ricin. As shown in Figure 3A, the RTA-induced luciferase synthesis rates in the co-incubated 100 and 200 μM RSMI-29 groups were approximately 1.5-fold and 2.5-fold higher, respectively, compared to the RTA-treated group alone. As shown in Figure 3B, similar experimental results were observed after the addition of ricin. Compared with the ricin control group, the synthesis rates of luciferase induced by ricin were increased by 1.5 times and 1.8 times in the 100 and 200 μM RSMI-29 treatment groups, respectively.

Ricin inhibits protein synthesis through its depurination activity [23]. Therefore, we further evaluated the effect of RSMI-29 on the rRNA depurination activity triggered by ricin. As shown in Figure 3C, compared to the DMSO control group, cells treated with 100 μM RSMI-29 showed a 28%, and cells treated with 200 μM RSMI-29 showed a 49%, decrease in rRNA depurination in a dose-dependent manner. These results indicate that RSMI-29 can attenuate the rRNA depurination caused by ricin and restore protein synthesis, thereby inhibiting the cytotoxicity of RTA or ricin.

### 2.4. RSMI-29 Directly Binds to RTA

**Figure 4:**
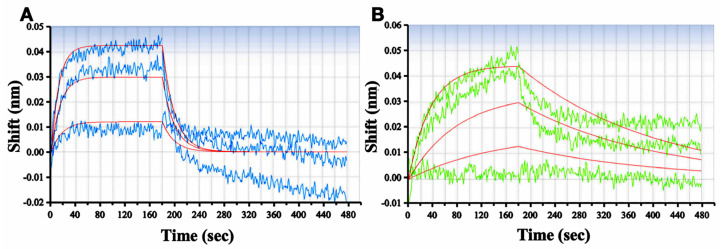
Binding kinetic activity of PTA and RSMI-29 to RTA. (**A**) Affinity kinetics of RTA with PTA was measured using Octet RED. The binding and dissociation curves of RTA with different concentrations of PTA (1, 3, and 5 μM) were detected by BLI; (**B**) affinity kinetics of RTA with RSMI-29 were measured using Octet RED. The binding and dissociation curves of RTA with different concentrations of RSMI-29 (3, 5, and 10 μM) were detected by BLI.

To verify the binding of RSMI-29 to RTA, the affinity constant between RSMI-29 and RTA was detected by BioLayer Interferometry (BLI), with the classic RTA inhibitor PTA as a positive control. Biotinylated RTA protein was immobilized on the sensor surface, and the binding and dissociation reactions were detected in the presence of different concentrations of PTA and RSMI-29. As shown in Figure 4A,B, molecular dynamics analysis showed that the affinity constant (KD) of RSMI-29 to RTA was 9.23 × 10^−7^ M, which was significantly lower than that of PTA (8.69 × 10^−6^ M). Furthermore, the association rate constant (Kon) and dissociation rate constant (Koff) parameters presented in Table 1 indicate that RSMI-29 binds to RTA faster than PTA, while the dissociation speed of RSMI-29 from RTA is slower than that of PTA. These results suggest that RSMI-29 can directly bind to RTA with high affinity.

### 2.5. Identify the Important Amino Acid Residues of RSMI-29 Interacting with RTA

**Figure 5:**
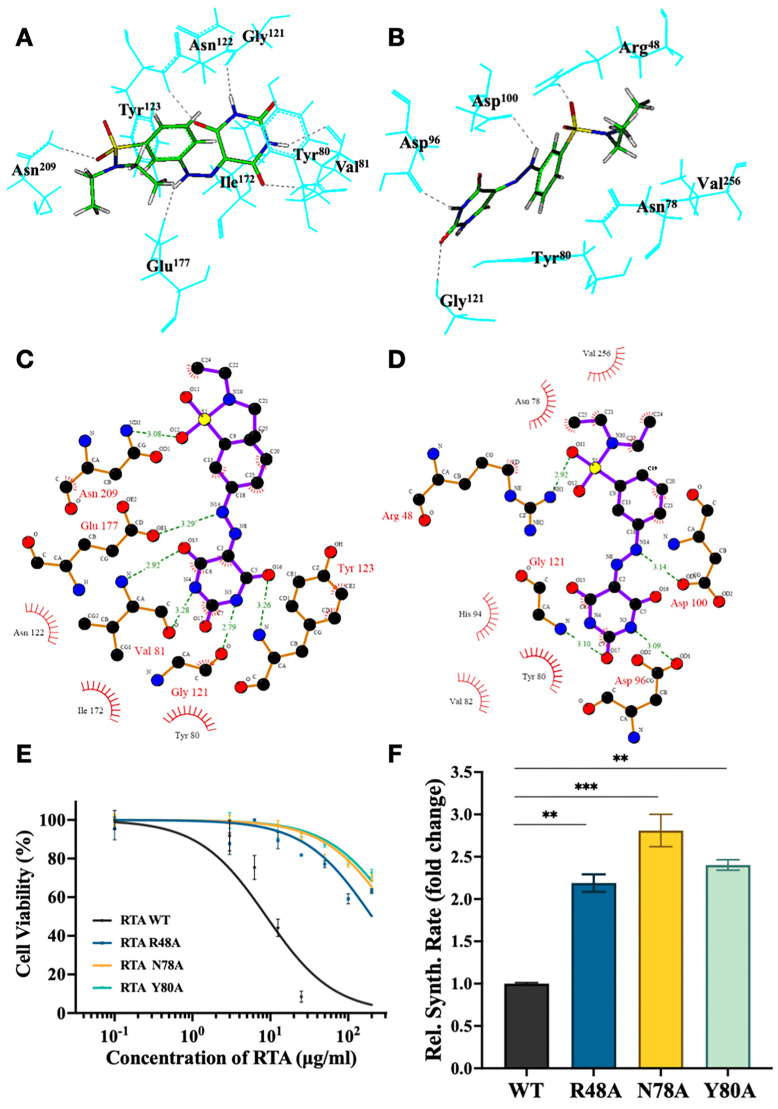
Theoretical prediction and identification of the interaction mode between RSMI-29 and RTA. (**A**,**B**) Three-dimensional structural models of the interaction between RSMI-29 and the primary pocket (**A**) and the secondary pocket (**B**) of RTA. (**C**,**D**) Two-dimensional structural models of the interaction between RSMI-29 and the primary pocket (**C**) and the secondary pocket (**D**) of RTA, with hydrogen bonds represented by dashed lines of varying lengths and hydrophobic contacts represented by arcs radiating from the ligand atoms that make contact. These figures were made with LIGPLOT [26]. (**E**) Cell survival curves for SP2/0 cells treated with RTA wild-type and its mutants for 24 h, followed by CCK8 assay. (**F**) Protein synthesis levels were measured using a cell-free protein synthesis reaction system with 20 μg/mL of wild-type RTA or its mutants. The luciferase synthesis level in the RTA WT group was set to 1, and the relative protein synthesis levels in the mutant groups are represented as mean ± SEM (** *p* < 0.01, *** *p* < 0.005 vs. RTA WT group, n = 3).

As described in the molecular docking analysis, we obtained both 3D and 2D stable complex conformations of RSMI-29 with the two active pockets of RTA, as displayed in Figure 5A–D. These results indicated that Arg^48^ (located in the secondary pocket), Asn^78^ (located in both pockets), and Tyr^80^ (located in both pockets) may be the key amino acid residues involved in the interaction between RSMI-29 and the secondary pocket of RTA. It is worth noting that Arg^48^ in the secondary pocket has not been reported as a key amino acid residue for RTA activity. To verify the functions of these three amino acid residues for RTA activity, RTA Arg^48^, Asn^78^, and Tyr^80^ mutants were constructed and evaluated regarding their activity in a cell-free protein synthesis system and cytotoxicity assay. As shown in Figure 5E,F, the cytotoxicity of RTA R48A was significantly reduced, and the protein synthesis rate was higher (approximately 2.2 times) than that of wild-type RTA, as expected. The phenotypes of RTA N78A and RTA Y80A were consistent with previous studies [24,25]. These findings suggest that Arg^48^, Asn^78^, and Tyr^80^ play important roles in the active region of RTA, which is consistent with theoretical predictions.

### 2.6. RSMI-29 Protects Mice against RICIN Intoxication

**Figure 6:**
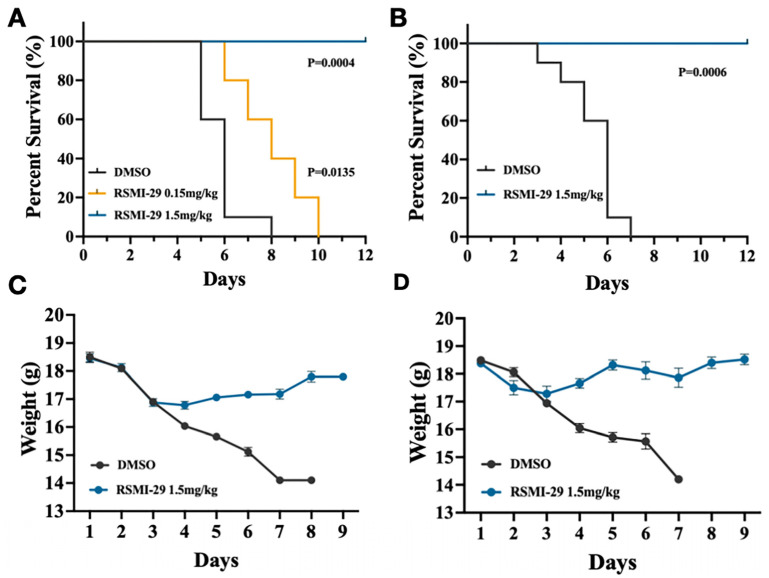
RSMI-29 protects mice against ricin intoxication. (**A**) Survival curves of C57BL/6J mice intraperitoneally injected with ricin 30 μg/kg and solvent DMSO or different doses of RSMI-29 simultaneously. At the end of the 12-day experiment, according to the log rank test, there were statistical differences in the survival curves of the RSMI-29 treatment groups compared to the DMSO group (RSMI-29 0.15 mg/kg group *p* = 0.0135; RSMI-29 1.5 mg/kg group *p* = 0.0004). (**B**) Survival curves of C57BL/6J mice intraperitoneally injected with ricin 35 μg/kg ricin and treated with DMSO or 1.5 mg/kg of RSMI-29 simultaneously (*p* = 0.0006). (**C**,**D**) Weight curves for each group of mice in the two experiments. (**C**,**D**) correspond to (**A**,**B**), respectively. The weight of mice is represented as mean ± SEM.

To evaluate whether RSMI-29 can protect mice from lethal ricin exposure, we conducted an intoxication experiment on C57BL/6J mice. As shown in Figure 6A, mice in the solvent group began to die on the 5th day after injection, and all mice had died on the 8th day, while mice in the low-dose group (0.15 mg/kg) started to die on the 6th day and all had died on the 10th day. Notably, all mice in the high-dose group (1.5 mg/kg) survived to the end of the experiment on day 12. As shown in Figure 6C, all mice exhibited a slight weight loss on days 2 and 3 after the intraperitoneal injection of twice the LD50 dose of ricin. However, from the 4th day, the body weight of the mice in the 1.5 mg/kg treatment group gradually returned to normal, while the mice in the solvent group continued to lose weight and displayed symptoms such as dry fur, lethargy, and diarrhea. Mice in the low-dose group (0.15 mg/kg) showed a similar trend of weight loss as those in the solvent control group, and there was no significant difference. Additionally, as shown in Figure 6B,D, the 1.5 mg/kg treatment group also protected mice from ricin intoxication at a higher dose, equivalent to 2.5 times the LD50.

### 2.7. RSMI-29 Reduces Ricin-Induced Liver, Spleen, Intestine, and Lung Injury in Mice

**Figure 7:**
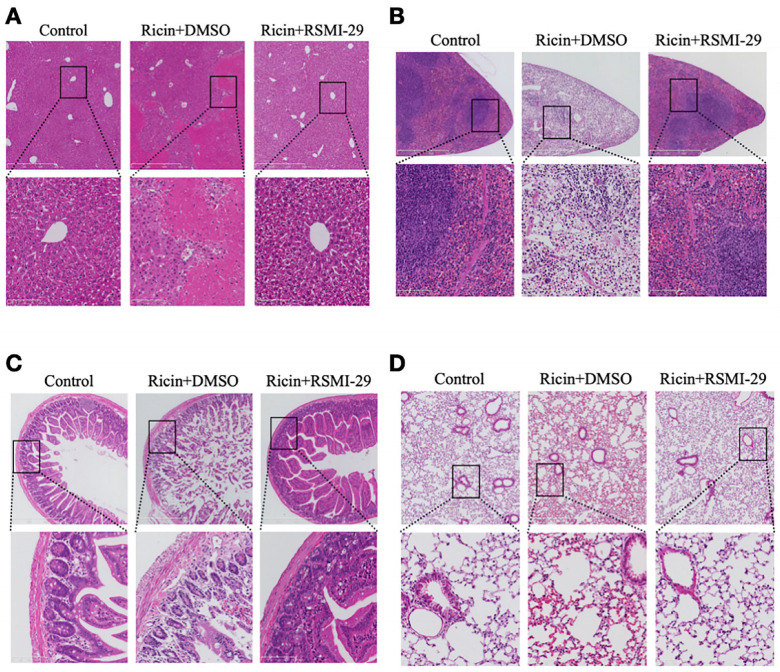
RSMI-29 reduces ricin-induced organ damage. (**A**–**D**) The livers (**A**), spleens (**B**), intestines (**C**), and lungs (**D**) of mice in the DMSO control group, 30 μg/kg ricin + DMSO group, and 30 μg/kg ricin + 1.5 mg/kg RSMI-29 group were fixed, sectioned, and stained with hematoxylin and eosin. Sections were scanned for magnification using slice scanning analyzer. The up were taken at a magnification of ×200, and the down were taken at a magnification of ×800, corresponding to the respective field of view (box).

To investigate the potential protective effect of RSMI-29 against ricin-induced organ damage, mice were dissected and histological analyses were conducted of mice in the DMSO control group, ricin + DMSO group, and ricin + RSMI-29 group, with a focus on organs sensitive to ricin toxicity [27,28,29]. During dissection, obvious white spots were observed in the livers of mice in the ricin + DMSO group, indicating obvious liver damage. Histological examination further confirmed this observation (Figure 7A), revealing scattered patch-like areas of necrosis in the livers of mice in the ricin + DMSO group, whereas the hepatic architecture of mice treated with RSMI-29 appeared normal, with intact hepatocytes, central veins, and hepatic sinusoids. Notably, the spleen volume of mice in the ricin + DMSO group was significantly reduced compared to the other two groups, accompanied by a paler color, suggesting possible ischemia. As shown in Figure 7B, the spleen cells of mice in the ricin + DMSO group displayed a loose arrangement, along with a notable decrease in lymphocytes in the white pulp and red pulp red blood cells, consistent with an ischemic phenotype. In contrast, mice treated with RSMI-29 exhibited abundant lymphoid follicles and germinal centers, indicating normal hematopoietic and immune function. The exposed mice showed symptoms such as diarrhea during the experiment, and the intestinal tissues were analyzed histologically. As depicted in Figure 7C, the intestinal villi of mice in the ricin + DMSO group exhibited obvious shedding into the intestinal lumen, along with a small amount of bleeding, and the submucosal basal cells displayed a loose and disordered wave pattern. Conversely, RSMI-29 significantly protected mice from ricin-induced intestinal injury, showing only a modest reduction in goblet cells. Furthermore, as shown in Figure 7D, no obvious pulmonary lesions were observed in the lungs of mice in each group, with only minimal alveolar bleeding observed in the exposed mice. Taken together, our histological analyses demonstrate that RSMI-29 exerts a protective effect against ricin-induced organ damage.

## 3. Discussion

Currently, no specific drugs are clinically available for the treatment of ricin intoxication. This has led to the pursuit of effective antidotes for ricin becoming an important research hotspot. The existing therapeutic options for ricin mainly consist of vaccines, neutralizing antibodies, and small-molecule inhibitors. Although the RiVax^®^ and RVEc^TM^ vaccines are currently undergoing phase I clinical trials [30,31], there are still limitations to their use. These vaccines demonstrate a good anti-ricin effect only on the premise of early prophylactic vaccination, and the activation of the immune response has a limited duration, requiring frequent booster injections [32]. Unfortunately, the vaccines are ineffective once ricin intoxication has already occurred. The use of neutralizing antibodies is also restricted by their macromolecular nature. When ricin exposure persists for a prolonged period of time, it becomes difficult for neutralizing antibodies to exert their therapeutic effects after the toxin has entered the cells [33]. Moreover, the production of vaccines and antibodies is complicated, expensive, and difficult to store, making the research and development of these two classes of anti-ricin drugs difficult to advance.

Small-molecule inhibitors have good cell membrane penetration ability and can exert anti-ricin effects inside and outside the cells. Therefore, small-molecule inhibitors have broad application prospects in both the prevention of ricin intoxication and treatment after ricin exposure [34]. Ricin consists of RTA and RTB linked by disulfide bonds. The galactose binding pocket of RTB is small and interacts weakly with lactose [35]. Due to the shallow polarity of galactose and the long distance between the two binding sites, the design of small-molecule inhibitors targeting RTB is very challenging, making RTA a more attractive target for structure-based drug design [36,37]. The active site of RTA is composed of two pockets, with the primary pocket catalyzing the depurination of A4324 and the secondary pocket accommodating adjacent guanine residues separated by Tyr^80^ [18]. Many studies have focused on designing inhibitors targeting the primary pocket [17,38,39]. In our study, our virtual screening strategy aimed to discover small-molecule compounds that can bind to both pockets of PTA, potentially with better anti-ricin pharmacological effects. The screen confirmed that RSMI-29 is a novel structure that can interact with both pockets of RTA. Furthermore, RSMI-29 has good drug-like characteristics, containing typical structures of known sulfonamides and barbiturates, which may reduce the risk of drug-development failure to some extent. In addition, we performed a molecular docking of RSMI-29 into RTA using the AutoDock VINA program (version 1.1.2). As shown in Figure S1, the cyan structure represents the binding pose of RMSI-29 obtained by the DOCK5.4.0 program, while the purple structure corresponds to that obtained by the AutoDock program. Encouragingly, the results from both docking programs are generally similar, with only minor differences in the binding posed in the primary pocket. We believe that it is helpful to cross-validate and enhance the accuracy of our hit prediction by using two different docking programs.

RSMI-29 exhibits significant antagonistic effects on the cytotoxicity induced by RTA or ricin, which are better than the previously reported small-molecule inhibitor PTA, due to its higher affinity for RTA. Moreover, RSMI-29 restored the RTA/ricin-induced inhibition of luciferase synthesis and weakened rRNA depurination in vitro. In the mice model, RSMI-29 also demonstrated the dose-dependent detoxification of ricin, with almost all mice showing resistance to ricin exposure (two times the LD50) when given a high dose of 1.5 mg/kg RSMI-29. Although no obvious toxic side effects were observed for RSMI-29 in vivo, further studies are necessary to investigate its metabolism, metabolites, and pharmacokinetics, as well as its anti-ricin activity prior to, or protective effects after, ricin exposure. Regarding our identified lead compound RSMI-29, as shown in Figure S2, we are aware that this compound contains a PAINs fragment [40]; further studies are ongoing to exclude the potential contribution of this fragment.

The interaction mode between RSMI-29 and RTA from molecular docking suggests that Arg^48^, Asn^78^, and Tyr^80^ may serve as key amino acid residues for interaction in the two pockets of ricin. The cytotoxicity and cell-free systems of RTA mutants further confirm the important role of these three amino acids for RTA activity. Asn^78^ and Tyr^80^ are amino acids located in the common region of the two active pockets. Although it has been reported that Asn^78^ and Tyr^80^ are essential for RTA toxicity [22,25], mainly for the primary active pocket, there are currently no reports highlighting the significance of Arg^48^ in the secondary active pocket for RTA activity. We also performed a molecular dynamics simulation and NMA analysis of RTA. As shown in Figure S3, the vibration amplitude is large and the effect is relatively significant for Arg^48^, while the other two amino acid residues have no obvious vibration. This result might suggest that Arg^48^ is important for protein function. We will express sufficient amounts of RTA mutants to determine the effect of different mutants on compound binding in our following studies. Further experiments are needed to verify whether RSMI-29 can bind to these amino acids of RTA, thus exerting its inhibitory effect through conformational changes. Moreover, further studies on the appropriate formulations and derivatives of RSMI-29 may enhance its solubility and improve its anti-ricin pharmacological effects, providing potential therapeutic approaches to drug development and the treatment of ricin intoxication. In addition, Abrin, another highly toxic type 2 RIP, has been reported to be structurally similar to ricin [41,42]. Therefore, further studies are needed to determine whether RSMI-29 has the potential to target and inhibit Abrin or other type 2 RIPs.

In summary, RSMI-29 is a novel small-molecule compound identified by rational virtual screening, and further studies have shown that RSMI-29 has a high affinity for RTA and can target both active pockets of RTA. In addition, our results showed that RSMI-29 attenuated rRNA depurination and the inhibition of protein synthesis induced by ricin, and exhibited significant anti-ricin effects both in vitro and in vivo, meaning that it may serve as an effective antidote against ricin in the future.

## 4. Materials and Methods

### 4.1. Chemical and Reagents

Ricin with a purity of over 95% was obtained from the Laboratory of Toxicant Analysis, Institute of Pharmacology and Toxicology, China, and its molecular weight was determined by MALDI-TOF/MS (Autoflex III Smartbeam, Bruker Daltonics Inc., Leipzig, Germany). PTA and dimethyl sulfoxide (DMSO) were purchased from Sigma-Aldrich (St. Louis, MO, USA). RSMI-29 was purchased from Specs (Narragansett, RI, USA). Both PTA and RSMI-29 were dissolved in DMSO and stored at −20 °C or −80 °C. The final concentration of DMSO did not exceed 0.1% (*v*/*v*) in cell experiments and 10% (*v*/*v*) in animal experiments. High-glucose Dulbecco’s Modified Eagle Medium (DMEM) cell culture medium and fetal bovine serum (FBS) were sourced from Thermo Fisher (Seattle, WA, USA). Phosphate-buffered saline (PBS) and penicillin-streptomycin were purchased from M&C Gene Technology (Beijing, China). All other chemicals were obtained from commercial sources of analytical grade.

### 4.2. Cell Line and Culture Condition

The mouse myeloma cell line SP2/0 was obtained from the American Type Culture Collection (Manassas, VA, USA). SP2/0 cells were maintained at 37 °C under 5% CO_2_ in DMEM supplemented with 10% FBS and 1% penicillin–streptomycin.

### 4.3. Animals Feeding

Specific pathogen-free, 8-week-old female C57BL/6J mice weighting 18–20 g were purchased from Charles River (Beijing, China). In brief, mice were fed with free access to standard diet in plastic cages at 21 ± 2 °C and maintained under a 12 h light–dark cycle. All procedures involving the use of animals were conducted in facilities fully accredited by the Association for Assessment and Accreditation of Laboratory Animal Care International (AAALAC International). All animal experimental protocols were reviewed and approved by the Institutional Animal Care and Use Committee of the Institute of Pharmacology and Toxicology (permit number IACUC-DWZX-2021-723).

### 4.4. Molecular Docking

The RTA catalytic region data file and 3D structure of RTA were retrieved from RCSB Protein Data Bank (PDB, https://www.rcsb.org, accessed on 22 June 2008). Heteroatoms such as water molecular and ligands were removed, while hydrogen and charges were added using SYBYL7.3. All instances of His were converted to Hid, and Cys was changed to Cyx. Docking calculations were performed using the flexible ligand docking program DOCK5.4.0 (provided by Kuntz Lab) based on the anchored search approach [43]. The standard docking approach was followed: (1) target preparation, (2) sphere set generation, (3) force field grid calculation, and (4) docking scoring. The top 2000 compounds with the highest energy score were clustered into a structurally diverse set based on their molecular fingerprints, and selected from individual groups using the selector program of SYBYL7.3 [44].

### 4.5. Expression and Purification of RTA and Its Mutants

The recombinant plasmid expressing RTA with pET32a as the vector was kindly provided by Prof. Jiannan Feng (Institute of Pharmacology and Toxicology, AMMS, AMS, Beijing, China) [45]. Plasmids expressing RTA mutants were amplified by PCR using the wild-type RTA plasmid as the template and the primers specified in Table 2. All these plasmids were transformed into *E. coli* BL21 (DE3) cells. A total of 400 μM Isopropyl-β-d-thiogalactopyranoside (IPTG) was added to the medium to induce expression, which was maintained at 20 °C. Bacteria were collected by centrifugation and ultrasonically crushed to obtain a crude extract. The His-tagged proteins were subsequently isolated using a His GraviTrap column (Cytiva, Marlborough, MA, USA) and concentrated using an ultrafiltration filter (Millipore, St. Louis, MO, USA) to obtain the target proteins [46].

### 4.6. Biolayer Interferometry (BLI)

The detection of biolayer interferometry was performed as previously described [47,48]. Prior to the experiment, purified RTA was dissolved in a dialysis buffer (PBS, pH 7.4) and biotinylated by adding EZ-Link NHS-LC-LC-Biotin for 30 min (Piece). Any unconjugated biotin was removed by column. The biosensors were pre-wet in a dialysis buffer for 15 min before use. For the binding affinity assay, the biosensors were loaded with biotinylated RTA for 15 min and then quenched with 5 μM EZ-Link Biocytin (Piece) for 1 min. The compound of interest was prepared in a serial dilution, either PTA (1, 3, and 5 μM) or RSMI-29 (3, 5, and 10 μM). The experimental procedure followed the instrument’s operation manual (Octet RED; ForteBio, San Francisco, CA, USA). Sensors without loading biotinylated RTA were used as the control to correct for baseline drift. All the experiments were performed at room temperature. Octet data analysis software (Fortebio version 11.0) was employed to analyze and process the data obtained from the octet instrument.

### 4.7. Cell-Free Luciferase Translation Assay

The reaction systems were prepared following the instructions provided for the TnT^®^ Coupled Reticulocyte Lysate System (Promega, Madison, WI, USA, L4610). Different concentrations of RSMI-29 (100 and 200 μM) and RTA/ricin were added to the systems, then mixed thoroughly and incubated for 90 min at 37 °C. After incubation, luciferin reaction buffer was added and luminescence was measured by BioTek Synergy H1 plate reader [49]. The data were presented as the fold change relative to RTA/ricin group, calculated as the ratio of Luc_drug+toxin_ and Luc_toxin_. Statistical analyses were carried out using Microsoft Excel (version 16.83) and GraphPad Prism 9.0.

### 4.8. Cytotoxicity Assay

The cytotoxicity assay was performed using a CCK-8 assay kit following the manufacturer’s protocol. SP2/0 cells were seeded at a density of 7000 cells per well in 96-well plates and incubated overnight. The cells were then treated with increasing concentrations of RTA (0.1, 2.5, 5, 10, 20, 30, 50, and 100 μg/mL) or ricin (0.005, 0.01, 0.04, 0.12, 0.37, 1.11, 3.33, 10, and 30 ng/mL) in the presence of different doses of inhibitor (PTA or RSMI-29). The inhibitor was added to 100 μL of medium without serum supplementation from 100% DMSO stocks to achieve the specified final concentrations for each treatment, and then added to the corresponding wells. After 24 h, cell viability was assessed by adding 10 μL of CCK8 to each well and incubating for approximately 2–4 h. The absorbance was measured by BioTek Synergy H1 plate reader at a test wavelength of 450 nm [50]. Viability was expressed as a percentage of control cells and EC50 was calculated using GraphPad Prism 9.0. The protection factor was calculated using the formula PF = EC50_toxin+drug_/EC50_toxin_, as described in a previous study [51].

### 4.9. Depurination Assay

The depurination assays were performed according to previously described methods [49,52]. SP2/0 cells were seeded in DMEM complete medium at a density of 2 × 10^5^ cells/mL in 12-well plates with 500 μL per well and incubated overnight. For depurination experiments, the complete medium was replaced with serum-free medium, which was prepared as the working solution for each experimental group. The cells were then co-incubated with different concentrations of RSMI-29 and 10 ng/mL ricin for 24 h. Total RNA was extracted from SP2/0 cells in each group using the RNAprep Pure Micro Kit (Tiangen, Beijing, China), either immediately or after storage at −80 °C. The extracted total RNA was reverse-transcripted into cDNA using the Prime Script RT Reagent Kit with gDNA Eraser (Takara, Tianjin, Japan). The q-PCR assays were performed on a Real-Time PCR System (BioRad CFX96, Hercules, CA, USA) using the Power SYBR Green Master Mix (Thermo Fisher Scientific, Seattle, WA, USA). Human 28S rRNA was used as the endogenous control, and the primer sequences for the q-PCR are listed in Table 2 [52]. RNA depurination levels were analyzed using the classic 2^−ΔΔCt^ method. The RNA depurination level in the ricin group was set as 100%, and relative depurination levels in the other groups were calculated accordingly.

### 4.10. In Vivo Experiments

Mice were intraperitoneally injected with normal saline solution containing 30 μg/kg or 35 μg/kg of ricin, along with DMSO or various concentrations of RSMI-29 (0.15 mg/kg and 1.5 mg/kg). The solvent control group was treated with saline and DMSO. Each mouse was injected with 10 μL of the solution per gram of body weight. Survival rates and body weights of the mice were recorded on a daily basis for up to 12 days after ricin intoxication.

### 4.11. Histological Analysis

After dissection, the liver, spleen, intestine, and lungs of mice were fixed in 4% neutral formaldehyde for 24 h. Then, the tissues were dehydrated in a series of gradient ethanol concentrations, cleared in xylene, embedded in paraffin, and sliced into 4 μm thick sections. After deparaffinization, standard hematoxylin and eosin staining was performed on the sections to assess histopathological changes in the tissues [53]. Tissue sections were scanned for magnification using a digital slice scanning analyzer nano zoomer 2.0 HT (Hamamatsu photonics, Berlin, Germany).

### 4.12. Statistical Analysis

Statistical analyses were conducted using GraphPad Prism 9.0 software (GraphPad Software, Inc., La Jolla, CA, USA). Student’s *t*-tests were performed to compare the two groups. One-way ANOVA was used for multiple group comparisons. Data are presented as the mean and standard error of the mean. The *p*-values were used to evaluate statistically significant differences between the tested groups (*p* < 0.05 was considered statistically significant).

## Figures and Tables

**Table 1:** Kon and Koff determined by the curves in Figure 4.

Compound	KD (M)	Kon (1/Ms)	Koff (1/s)
PTA	8.68 × 10^−6^	5.92 × 10^3^	5.14 × 10^−2^
RSMI-29	9.23 × 10^−7^	8.32 × 10^3^	7.68 × 10^−3^

**Table 2:** Primers used for PCR and qPCR.

	Forward Primers (5′-3′)	Reverse Primers (5′-3′)
RTA R48A	GTTGCCAAACGCAGTTGGTTTGCCTATAAACC	ACCAACTGCGTTTGGCAACACTGGTATTTC
RTA N78A	GGATGTCACCGCTGCATATGTGGTCGGCTAC	CATATGCAGCGGTGA CATCCAGGGCTAATG
RTA Y80A	CACCAATGCAGCTGTGG TCGGCTACC	AGCCGACCACATATG CATTGGTGACATCCA
Human depurinated rRNA	TGCCATGGTAATCCTGCT CAGTA	TCTGAACCTGCGGTT CCACA
Human 28S rRNA	GATGTCGGCTCTTCCTAT CATTGT	CCAGCTCACGTTCCC TATTAGTG

## Data Availability

Data are contained within the article.

## Supplementary Materials

The following supporting information can be downloaded at: https://www.mdpi.com/article/10.3390/molecules29071435/s1, Figure S1. The binding modes of inhibitor RMSI-29 in primary pocket of RTA (left) and in the secondary pocket (right) obtained from both DOCK 5.4.0 (colored in cyan) and AutoDock VINA program (colored in purple). Figure S2. The structure of lead compound #29 with the PAINs fragment highlighted in red box. Figure S3. Normal mode analysis of RTA residues.

## References

[1] Sowa-Rogozińska N., Sominka H., Nowakowska-Gołacka J., Sandvig K., Słomińska Wojewódzka M. (2019). Intracellular Transport and Cytotoxicity of the Protein Toxin Ricin. Toxins.

[2] Brunka Z., Ryl J., Brushtulli P., Gromala D., Walczak G., Zięba S., Pieśniak D., Anand J.S., Wiergowski M. (2022). Selected Political Criminal Poisonings in the Years 1978–2020: Detection and Treatment. Toxics.

[3] Janik E., Ceremuga M., Saluk-Bijak J., Bijak M. (2019). Biological Toxins as the Potential Tools for Bioterrorism. Int. J. Mol. Sci..

[4] Polito L., Bortolotti M., Battelli M.G., Calafato G., Bolognesi A. (2019). Ricin: An Ancient Story for a Timeless Plant Toxin. Toxins.

[5] Abbes M., Montana M., Curti C., Vanelle P. (2021). Ricin poisoning: A review on contamination source, diagnosis, treatment, prevention and reporting of ricin poisoning. Toxicon.

[6] Franke H., Scholl R., Aigner A. (2019). Ricin and Ricinus communis in pharmacology and toxicology-from ancient use and “Papyrus Ebers” to modern perspectives and “poisonous plant of the year 2018”. Naunyn Schmiedebergs Arch. Pharmacol..

[7] Spooner R.A., Lord J.M. (2015). Ricin trafficking in cells. Toxins.

[8] Walsh M.J., Dodd J.E., Hautbergue G.M. (2015). Ribosome-inactivating proteins: Potent poisons and molecular tools. Virulence.

[9] Fang E.F., Ng T.B., Shaw P.C., Wong R.N. (2011). Recent progress in medicinal investigations on trichosanthin and other ribosome inactivating proteins from the plant genus Trichosanthes. Curr. Med. Chem..

[10] Gaylord S.T., Dinh T.L., Goldman E.R., Anderson G.P., Ngan K.C., Walt D.R. (2015). Ultrasensitive Detection of Ricin Toxin in Multiple Sample Matrixes Using Single-Domain Antibodies. Anal. Chem..

[11] Yu H., Li S., Xu N., Liu W. (2022). Ricin toxin and its neutralizing antibodies: A review. Toxicon.

[12] Sarkes D.A., Hurley M.M., Stratis-Cullum D.N. (2016). Unraveling the Roots of Selectivity of Peptide Affinity Reagents for Structurally Similar Ribosomal Inactivating Protein Derivatives. Molecules.

[13] Rasetti-Escargueil C., Avril A. (2023). Medical Countermeasures against Ricin Intoxication. Toxins.

[14] Yan X., Hollis T., Svinth M., Day P., Monzingo A.F., Milne G.W., Robertus J.D. (1997). Structure-based identification of a ricin inhibitor. J. Mol. Biol..

[15] Pruet J.M., Jasheway K.R., Manzano L.A., Bai Y., Anslyn E.V., Robertus J.D. (2011). 7-Substituted pterins provide a new direction for ricin A chain inhibitors. Eur. J. Med. Chem..

[16] Saito R., Pruet J.M., Manzano L.A., Jasheway K., Monzingo A.F., Wiget P.A., Kamat I., Anslyn E.V., Robertus J.D. (2013). Peptide-conjugated pterins as inhibitors of ricin toxin A. J. Med. Chem..

[17] Pruet J.M., Saito R., Manzano L.A., Jasheway K.R., Wiget P.A., Kamat I., Anslyn E.V., Robertus J.D. (2012). Optimized 5-membered heterocycle-linked pterins for the inhibition of Ricin Toxin A. ACS Med. Chem. Lett..

[18] Wahome P.G., Robertus J.D., Mantis N.J. (2012). Small-molecule inhibitors of ricin and Shiga toxins. Curr. Top. Microbiol. Immunol..

[19] França T.C.C., Botelho F.D., LaPlante S.R. (2022). Theoretical Investigation of Repurposed Drugs Potentially Capable of Binding to the Catalytic Site and the Secondary Binding Pocket of Subunit A of Ricin. ACS Omega.

[20] Huang N., Nagarsekar A., Xia G., Hayashi J., MacKerell A.D. (2004). Identification of non-phosphate-containing small molecular weight inhibitors of the tyrosine kinase p56 Lck SH2 domain via in silico screening against the pY + 3 binding site. J. Med. Chem..

[21] Peng H., Huang N., Qi J., Xie P., Xu C., Wang J., Yang C. (2003). Identification of novel inhibitors of BCR-ABL tyrosine kinase via virtual screening. Bioorg. Med. Chem. Lett..

[22] Mishra V., Siva Prasad C.V. (2011). Ligand based virtual screening to find novel inhibitors against plant toxin Ricin by using the ZINC database. Bioinformation.

[23] Cong D., Li Y., Ludford P.T., Tor Y. (2022). Isomorphic Fluorescent Nucleosides Facilitate Real-Time Monitoring of RNA Depurination by Ribosome Inactivating Proteins. Chemistry.

[24] Kim Y., Robertus J.D. (1992). Analysis of several key active site residues of ricin A chain by mutagenesis and X-ray crystallography. Protein Eng..

[25] Ready M.P., Kim Y., Robertus J.D. (1991). Site-directed mutagenesis of ricin A-chain and implications for the mechanism of action. Proteins.

[26] Wallace A.C., Laskowski R.A., Thornton J.M. (2015). LIGPLOT: A program to generate schematic diagrams of protein-ligand interactions. Protein Eng..

[27] Sapoznikov A., Gal Y., Evgy Y., Aftalion M., Katalan S., Sabo T., Kronman C., Falach R. (2021). Intramuscular Exposure to a Lethal Dose of Ricin Toxin Leads to Endothelial Glycocalyx Shedding and Microvascular Flow Abnormality in Mice and Swine. Int. J. Mol. Sci..

[28] Roy C.J., Song K., Sivasubramani S.K., Gardner D.J., Pincus S.H. (2012). Animal models of ricin toxicosis. Curr. Top. Microbiol. Immunol..

[29] Sapoznikov A., Rosner A., Falach R., Gal Y., Aftalion M., Evgy Y., Israeli O., Sabo T., Kronman C. (2019). Intramuscular Ricin Poisoning of Mice Leads to Widespread Damage in the Heart, Spleen, and Bone Marrow. Toxins.

[30] Roy C.J., Ehrbar D., Van Slyke G., Doering J., Didier P.J., Doyle-Meyers L., Donini O., Vitetta E.S., Mantis N.J. (2022). Serum antibody profiling identifies vaccine-induced correlates of protection against aerosolized ricin toxin in rhesus macaques. NPJ Vaccines.

[31] Montgomery V.A., Lindsey C.Y., Smith L.A., Webb R.P. (2021). Development of an o-pthalaldehyde (OPA) assay to measure protein content in Ricin Vaccine *E. coli* (RVEc™). Vaccine.

[32] Botelho F.D., Franca T.C.C., LaPlante S.R. (2024). The Search for Antidotes Against Ricin. Mini Rev. Med. Chem..

[33] Vance D.J., Rudolph M.J., Davis S.A., Mantis N.J. (2023). Structural Basis of Antibody-Mediated Inhibition of Ricin Toxin Attachment to Host Cells. Biochemistry.

[34] Zhao X., Li H., Li J., Liu K., Wang B., Wang Y., Li X., Zhong W. (2020). Novel small molecule retrograde transport blocker confers post-exposure protection against ricin intoxication. Acta Pharm. Sin. B.

[35] Jasheway K., Pruet J., Anslyn E.V., Robertus J.D. (2011). Structure-based design of ricin inhibitors. Toxins.

[36] Goto M., Sakamoto N., Higashi S., Kawata R., Nagatsu K., Saito R. (2023). Crystal structure of ricin toxin A chain complexed with a highly potent pterin-based small-molecular inhibitor. J. Enzym. Inhib. Med. Chem..

[37] Chaves E.J.F., Gomes da Cruz L.E., Padilha I.Q.M., Silveira C.H., Araujo D.A.M., Rocha G.B. (2022). Discovery of RTA ricin subunit inhibitors: A computational study using PM7 quantum chemical method and steered molecular dynamics. J. Biomol. Struct. Dyn..

[38] Kumar R.B., Suresh M.X. (2012). A computational perspective of molecular interactions through virtual screening, pharmacokinetic and dynamic prediction on ribosome toxin A chain and inhibitors of Ricinus communis. Pharmacogn. Res..

[39] Bai Y., Watt B., Wahome P.G., Mantis N.J., Robertus J.D. (2010). Identification of new classes of ricin toxin inhibitors by virtual screening. Toxicon.

[40] Capuzzi S.J., Muratov E.N., Tropsha A. (2017). Phantom PAINS: Problems with the Utility of Alerts for Pan-Assay INterference CompoundS. J. Chem. Inf. Model..

[41] Phatak P., Chauhan V., Dhaked R.K., Pathak U., Saxena N. (2021). E-N-(2-acetyl-phenyl)-3-phenyl-acrylamide targets abrin and ricin toxicity: Hitting two toxins with one stone. Biomed. Pharmacother..

[42] Sapoznikov A., Gal Y., Alcalay R., Evgy Y., Sabo T., Kronman C., Falach R. (2022). Characterization of Lung Injury following Abrin Pulmonary Intoxication in Mice: Comparison to Ricin Poisoning. Toxins.

[43] Moustakas D.T., Lang P.T., Pegg S., Pettersen E., Kuntz I.D., Brooijmans N., Rizzo R.C. (2006). Development and validation of a modular, extensible docking program: DOCK 5. J. Comput. Aided Mol. Des..

[44] Miteva M.A., Lee W.H., Montes M.O., Villoutreix B.O. (2005). Fast structure-based virtual ligand screening combining FRED, DOCK, and Surflex. J. Med. Chem..

[45] Wang S.T., Hu M.R., Guo J.W., Feng J.N., Shen B.F. (2005). Fusion expression and purification of recombinant ricin A-chain. Chin. J. Cell. Mol. Immunol..

[46] Xu R., Li D., Peng J., Fang J., Zhang L., Liu L. (2016). Cloning, expression and antioxidant activity of a novel collagen from *Pelodiscus sinensis*. World J. Microbiol. Biotechnol..

[47] Dong N., Luo L., Wu J., Jia P., Li Q., Wang Y., Gao Z., Peng H., Lv M., Huang C. (2015). Monoclonal antibody, mAb 4C13, an effective detoxicant antibody against ricin poisoning. Vaccine.

[48] Liu S., Liu Z., Piao C., Zhang Z., Kong C., Yin L., Liu X. (2022). Flavokawain A is a natural inhibitor of PRMT5 in bladder cancer. J. Exp. Clin. Cancer Res..

[49] Zhou Y., Li X.P., Kahn J.N., Tumer N.E. (2018). Functional Assays for Measuring the Catalytic Activity of Ribosome Inactivating Proteins. Toxins.

[50] Stechmann B., Bai S.K., Gobbo E., Lopez R., Merer G., Pinchard S., Panigai L., Tenza D., Raposo G., Beaumelle B. (2010). Inhibition of retrograde transport protects mice from lethal ricin challenge. Cell.

[51] Chen Q., Han H., Lin F., Yang L., Feng L., Lai X., Wen Z., Yang M., Wang C., Ma Y. (2022). Novel shikonin derivatives suppress cell proliferation, migration and induce apoptosis in human triple-negative breast cancer cells via regulating PDK1/PDHC axis. Life Sci..

[52] Li X.P., Harijan R.K., Cao B., Kahn J.N., Pierce M., Tsymbal A.M., Roberge J.Y., Augeri D., Tumer N.E. (2021). Synthesis and Structural Characterization of Ricin Inhibitors Targeting Ribosome Binding Using Fragment-Based Methods and Structure-Based Design. J. Med. Chem..

[53] Lu J., Fang B., Huang Y., Tao S., Sun B., Guan S., Jin Y. (2018). Epigallocatechin-3-gallate protects against 1,3-dichloro-2-propanol-induced lipid accumulation in C57BL/6J mice. Life Sci..

