# Supplementary material for: Identification and Biological Evaluation of a Novel Small-Molecule Inhibitor of Ricin Toxin
Source: Molecules. 2024 Mar 22;29(7):1435. doi: 10.3390/molecules29071435 (PMC11012547; doi:10.3390/molecules29071435)
Supplement: Supplementary file 1 [file molecules-29-01435-s001.zip › molecules-2910682-supplementary.pdf]

Supplementary Materials

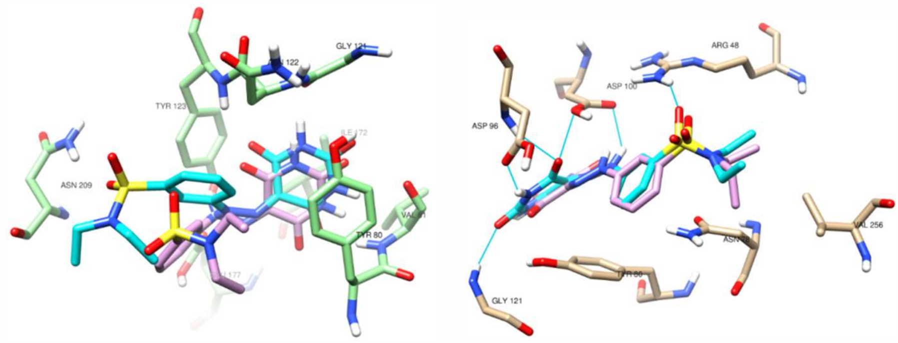

Figure S1. The binding modes of inhibitor RMSI-29 in primary pocket of RTA (left) and in the secondary pocket (right) obtained from both DOCK 5.4.0 (colored in cyan) and AutoDock VINA program (colored in purple).

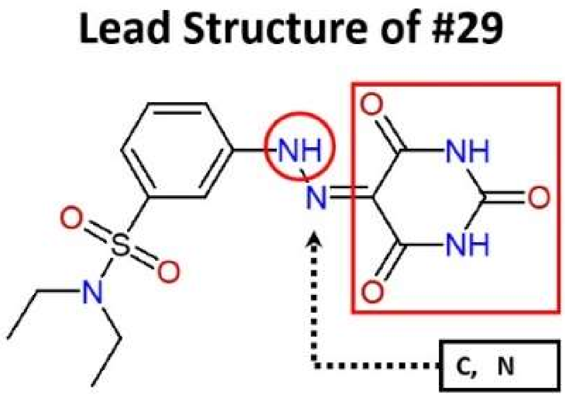

Figure S2. The structure of lead compound #29 with the PAINs fragment highlighted in red box.

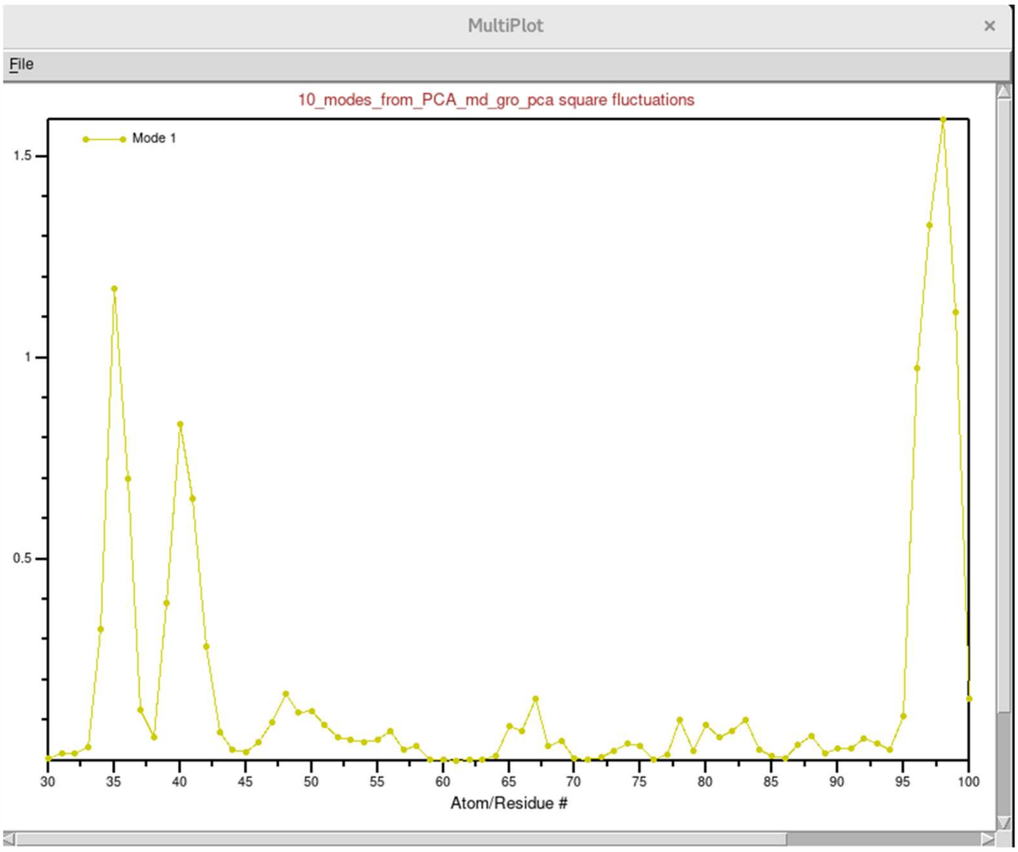

Figure. S3 Normal mode analysis of RTA residues
